# NutriChem 2.0: exploring the effect of plant-based foods on human health and drug efficacy

**DOI:** 10.1093/database/bax044

**Published:** 2017-06-11

**Authors:** Yueqiong Ni, Kasper Jensen, Irene Kouskoumvekaki, Gianni Panagiotou

**Affiliations:** 1 Systems Biology & Bioinformatics Group, School of Biological Sciences, The University of Hong Kong, Pokfulam Road, Hong Kong; 2 NNIT A/S, Ostmarken 3A, Soborg DK 2860, Denmark; 3 Department of Bioinformatics, Technical University of Denmark, Kemitorvet, Building 208, DK-2800 Lyngby, Denmark; 4 Department of Systems Biology and Bioinformatics, Leibniz Institute for Natural Product Research and Infection Biology, Hans Knoll Institute (HKI), Beutenbergstraße 11a, 07745 Jena, Germany

## Abstract

NutriChem is a database generated by text mining of 21 million MEDLINE abstracts that links plant-based foods with their small molecule components and human health effect. In this new, second release of NutriChem (NutriChem 2.0) we have integrated information on overlapping protein targets between FDA-approved drugs and small compounds in plant-based foods, which may have implications on drug pharmacokinetics and pharmacodynamics. NutriChem 2.0 contains predicted interactions between 428 drugs and 339 foods, supported by 107 jointly targeted proteins. Chemical bioactivity data were integrated, facilitating the comparison of activity concentrations between drugs and phytochemicals. In addition, we have added functionality that allows for user inspection of supporting evidence, the classification of food constituents based on KEGG “Phytochemical Compounds”, phytochemical structure output in SMILES and network output in both static figure and Cytoscape-compatible xgmml format. The current update of NutriChem moves one step further towards a more comprehensive assessment of dietary effects on human health and drug treatment.

**Database URL**: http://sbb.hku.hk/services/NutriChem-2.0/

## Introduction

As one of the most dynamic and common environmental factors, diet can affect human health in a variety of ways (1–4). Plant-based diet (i.e. fruits and vegetables) is often regarded as “healthy diet”. To provide a systematic overview and to create a platform for the study of the complex composition of plant-based foods (referred to as “foods” below) and their medicinal value, we previously developed NutriChem 1.0 by text mining of 21 million MEDLINE abstracts (5). That release of NutriChem contains the food-compound pairs between 1772 plant-based foods and 7898 phytochemicals, as well as the food-disease associations between 1066 plant-based foods and 751 diseases.

However, plant-based foods contain a wide range of phytochemicals that may also interfere with drug pharmacodynamic and/or pharmacokinetic processes (6, 7). Several well-known drug–food interactions, either through anecdotal experience or scientific research exist. For example, orange juice is known to decrease the intestinal absorption of celiprolol, an antihypertensive drug (8); sesame seeds negatively interfere with tamoxifen affecting the tumor-inhibitory efficiency of the drug (9); cranberry juice may inhibit CYP2C9 activity, the main metabolizing enzyme for warfarin (10). In addition, many flavonoids including quercetin, catechins and kaempferol were reported to modulate the absorption and bioavailbility of certain drugs. For example, drugs that are P-glycoprotein substrates can be affected by phytochemicals present in plant-based foods such as green tea and St. John's wort (11). Drug bioavailability can also be altered by diet leading to unexpected side effects and toxicity. Researchers and pharmacists have also categorized the interactions between drugs and general nutrients into different types, according to the physiological sequence of drug administration and interaction mechanism (7). As we have previously demonstrated (12), therapeutic interventions for most disease classes could be potentially influenced by diet and dietary molecules. An easy-to-access resource of such interactions could serve as a guideline for medical doctors about particular foods that should be avoided under certain medication. Although other efforts exist, such as DrugBank (13) that describes drug–food interactions and the associated outcome, where such information is available, ignoring the rich natural compound content inevitably underestimates the magnitude of potential drug–food interactions.

Here, we present the second release of NutriChem, accessible at http://sbb.hku.hk/services/NutriChem-2.0. The major novel feature of the new release is the mapping of drug–food interactions based on overlapping protein targets between FDA-approved drugs and plant-based foods, with the extensive and complex phytochemical content of foods taken into account. Moreover, we have added functionality to export compound structures in SMILES and have incorporated compound classifications in the query outputs. NutriChem-2.0 offers a platform for examining disease–food and drug–food interactions and supports a more thorough assessment of dietary influence on human health and therapy.

## Implementation

### Data ontologies

Using text-mining of 21 million MEDLINE abstracts, we previously associated 1772 plant-based foods with their 7898 small molecule components and 1066 foods with 751 human diseases, and integrated them into NutriChem (5). To infer food–drug interactions, all food–compound associations from NutriChem-1.0 were used. FDA-approved small-molecule drugs and drug names were retrieved from DrugBank V4.5.0 (13). Drugs were mapped to indicated diseases and disease classes using the Therapeutic Target Database (TTD) (14) and the Disease Ontology (DO) database (15), with “disease class” based on the third level of disease hierarchy in DO.

### Drug–food interaction data

**Figure 1.**
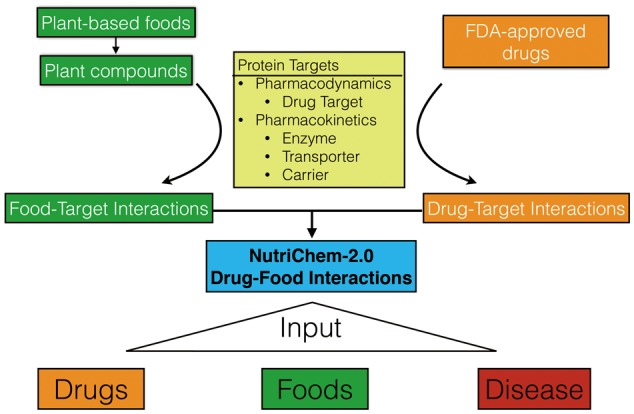
The flowchart of the “Drug–Food Interactions” section of NutriChem-2.0 and the three different query options (drugs, foods and disease).

Both drug–protein and dietary compound–protein interactions were retrieved as previously described (12), further expanded to include ChEMBL (16) bioactivity values of K_i_, K_d_, IC_50_ and EC_50_. The ChEMBL-normalized values (pChEMBL, i.e. negative log10 transformation) of such bioactivities were used to facilitate comparisons and integration of different measurement types. A dietary compound–protein interaction was included only if the binding bioactivity was up to 20% lower than the minimum bioactivity of drugs targeting the same protein. For example, for a target with drug bioactivity values in the range 3–4 µM, we have included all food compound bioactivities up to 4.8 µM. The interactions between FDA-approved drugs and plant-based foods were created, on the basis of overlapping interacting protein partners between drugs and food compounds (Figure 1), as well as the food-compound associations from NutriChem-1.0. Disease annotations to drug-food interactions were retrieved, when available, from TTD, in contrast with our previous work, where disease names were mapped from drug protein targets (12). According to the disease hierarchy in DO, we further associated drug–food interactions to a disease class, such as cancer and cardiovascular.

### Other updates in NutriChem-2.0

Apart from the novel drug-food interaction section, we also updated several aspects in “plant–compound search”. We made phytochemical structures downloadable in SMILES format (at the bottom-right corner after searches by foods with “Food-Disease Associations” selected), allowing users to perform a range of chemoinformatics analyses including, but not limited to, structural similarity comparisons and cross-reference to other databases. To facilitate a better characterization of food constituents, we added hierarchical compound classification information based on KEGG “Phytochemical Compounds” (http://www.genome.jp/kegg-bin/get_htext?br08003.keg). Examples include flavonols and flavones that belong to flavonoids, as well as carotenoids categorized into terpenoids. Such information is now available when users query a food in “Food-Disease Association” section of NutriChem.

## Database description

In addition to the data already incorporated in NutriChem-1.0, the new NutriChem-2.0 includes 14 662 drug–food interactions covering 107 proteins jointly targeted by 428 drugs and 339 foods. It further categorizes drug–food interactions in 59 diseases and 19 general disease classes, among which nervous, cardiovascular and musculoskeletal system diseases appear to be the most vulnerable to drug-food interactions.

The “Food Interactions” section in DrugBank for the anticoagulant drug warfarin has the following information: “Avoid St. John's Wort”; “Consult your doctor before ingesting large amounts of dietary Vitamin K (e.g. from green leafy vegetables)”; “Limit garlic, ginger, gingko and horse chestnut” (http://www.drugbank.ca/drugs/DB00 682). NutriChem 2.0 zooms at the molecular layer of the aforementioned interaction and supports the anecdotal knowledge with literature evidence for two jointly targeted proteins (P02768 and P11712) between warfarin and multiple bioactive compounds in St John’s wort (e.g. kaempferol); parsley (whose interaction with warfarin has also been reported previously) (10) and ginkgo (*Ginkgo biloba*) also appear in the list of warfarin-interacting foods with appropriate molecular-based support.

The homepage of NutriChem-2.0 has been designed to accommodate searches by food, disease, compound or drug. A detailed instruction on NutriChem-2.0 usage is available on the server. Users can choose among one of the following two query options:

1. The updated “Food-Disease Associations” section that contains all the functionality of NutriChem-1.0 accepts food, disease and compound queries and returns plant–disease associations and/or plant–compound associations. In addition, it provides experimental data on phytochemical–protein interactions and predicted compound–disease associations. This section links the chemical space of plant-based foods with human disease phenotypes and offers a platform to explore the medicinal value of foods.
2. The new “Drug-Food Interactions” query serves as the major update, as described in detail below. It accepts three types of queries as input (Figure 1): (a) drug query, where a drug name or DrugBank ID may be used; (b) food query, using a plant-based food name or corresponding NCBI Taxonomy ID; (c) disease query, where users may either provide a disease name (or corresponding IDs in Disease Ontology database) or select a disease class name. To the best of our knowledge, this release of NutriChem is the first resource for performing such food-centric search of drug–food interactions. Before submitting a query, the user has the option to define the number of jointly targeted proteins required for an edge (i.e. one drug–food interaction pair) and the number of edges allowed to a node in the generated CytoScapeWeb network (http://cytoscapeweb.cytoscape.org). By default, we use a minimum of one protein for an edge (ranging from 1 to 10) and a maximum of 15 edges for a node (ranging from 5 to “All edges”). The threshold on proteins applies to both network visualization and result table, while the limit on number of edges applies only to the network. It is, however, not recommended to show all edges because this will consume system resources, when massive amount of data are returned. A default search returns all retrieved drug–food interactions, as well as the number of proteins jointly targeted by the drug and the phytochemicals. The user can zoom in the interactions involving specific drug-interacting protein types (drug targets, enzymes, transporters and carriers) or return to the homepage and submit a new query. The drug–food interactions affecting drug targets may interfere with drug pharmacodynamics, while those involving enzymes, transporters and carriers can influence the pharmacokinetic process.

## Case study

**Figure 2.**
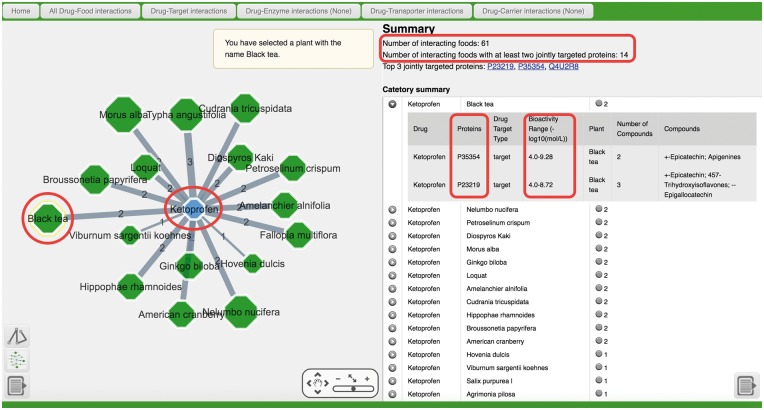
The example output page when using the drug Ketoprofen as a query of Drug–Food Interaction section.

Ketoprofen (DrugBank ID: DB01009) is a drug indicated for rheumatoid arthritis, osteoarthritis and alleviation of moderate pain. Using it to query the “Drug–Food Interaction” section of NutriChem returns, by default, a list of 61 unique plant-based foods (ordered by the number of jointly targeted proteins) that may affect its efficacy (bottom right, Figure 2). The summary section (top right) of the web page shows that 14 (23%) of them appear to contain compounds that exhibit biological activity against two disease-relevant proteins. Clicking on the “Drug Target Interactions” button at the top displays 61 foods, where the drug–food interactions are based on overlapping “drug targets”. Notably, most of them are found to affect pharmacodynamics by interfering with Ketoprofen targets such as prostaglandin G/H synthase 1 (P23219) and prostaglandin G/H synthase 2 (P35354), whereas almost no interactions are related to drug pharmacokinetics, with the exception of one interaction between narrowleaf cattail (*Typha angustifolia*) and the drug transporter solute carrier family 22 member 6 (Q4U2R8). Clicking on one drug–food pair in the table or one particular edge in the network will display detailed information, including drug names, protein targets, types of protein targets, drug–target bioactivity range, food names, the numbers and names of plant compounds for each food–protein interaction. Within the network, the edge width reflects the number of proteins for each drug-food pair. The size of food node reflects the total number of interaction pairs between compounds in the involved food and protein targets jointly targeted with Ketoprofen. If desired, users may also manipulate the network or zoom in and out using the toolbox.

Black tea has two overlapping protein targets with Ketoprofen and clicking on this pair reveals a total of 5 phytochemical–protein interactions, as also indicated by the medium node size in the network (Figure 2). The user may click on the target, drug or food name, in order to be redirected to UniProt, DrugBank and NCBI Taxonomy Browser, respectively. Moreover, clicking on the “Number of Compounds” field will show the phytochemicals (i.e. epigallocatechin and apigenin in Black tea) responsible for the underlying biological activity, and, hence, for the potential drug–food interaction, as well as PubMed evidence for the food–compound association, their ChEMBL compound records, the compound–target bioactivities and the option to export compound structures in SMILES format (bottom-right corner of web page). The full output list and the network are downloadable using the “Export table” and “Export network” (as PDF or XGMML) button (Figure 2), which can be opened and edited using text editor, image processor (for PDF) or Cytoscape (17) (for XGMML), respectively.

**Figure 3.**
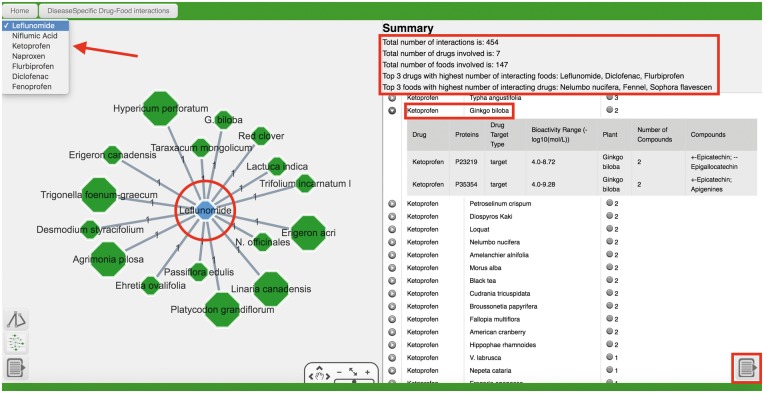
Using rheumatoid arthritis as a disease query of NutriChem-2.0 Drug–Food Interaction section.

Querying for rheumatoid arthritis (or “DOID: 7148”)—the disease indication of Ketoprofen—in the “search by disease” field and selecting “Drug–Food Interactions” takes the user to an extensive collection of disease-specific drug–food interactions, covering seven different drugs, including Ketoprofen (Figure 3). In the results table, the different drugs are listed with corresponding drug-food interactions ordered by the number of proteins. For clarity in network visualization, only the drug with the highest number of interacting foods (Leflunomide in this case) is shown by default, together with the foods that it potentially interacts with. Users may switch to the network for another drug of interest, using the dropdown menu at the upper-left corner (Figure 3). Some plant-based foods or herbs, for example ginkgo, black tea and Japanese honeysuckle (*Lonicera japonica*), are found to potentially interfere with the pharmacological properties of all seven drugs. Last but not least, selecting as query the general disease class “Musculoskeletal System Disease” that rheumatoid arthritis belongs to, returns in total interactions between 16 drugs and 174 plant-based foods.

## Conclusion

Although sporadic information on drug–food interactions has been deposited in databases such as DrugBank (13), clinical management of most interactions are largely based on anecdotal experience and uncontrolled observations (7). There is still a lack of systematic and molecular-level collection and prediction of such data. In NutriChem-2.0 database, we incorporated information on overlapping targets between drugs and food constituents that may have implications on drug pharmacodynamic and pharmacokinetic process. This update of NutriChem, integrating all together diet–drug–disease interactions, moves one step further towards a more comprehensive assessment of dietary effects on human health and drug treatment. Pharmacists and nutritionists can access such interactions easily and take actions accordingly. Having said that, we should bear in mind that experimental evidence of a drug and a phytochemical sharing a protein target is not a proof for the presence of a drug–food interaction but rather consists an indication that should be further established via experimental and clinical validations. In line with this, the incorporation of confidence scores based on the availability of support from literature or patient records may serve as future update of NutriChem.

## Funding

No funding has been received for this work.

*Conflict of interest*. None declared.
